# Work-related burnout and its associated factors among kindergarten teachers: a multi-center cross-sectional study in Ethiopia

**DOI:** 10.3389/fpubh.2024.1453504

**Published:** 2025-01-21

**Authors:** Anmut Endalkachew Bezie, Girum Tareke Zewude, Amensisa Hailu Tesfaye, Asmare Asrat Yirdaw, Alebachew Bitew Abie, Giziew Abere

**Affiliations:** ^1^Department of Occupational Health and Safety, College of Medicine and Health Sciences, Wollo University, Dessie, Ethiopia; ^2^Department of Psychology, Wollo University, Dessie, Ethiopia; ^3^Department of Environmental and Occupational Health and Safety, Institute of Public Health, College of Medicine and Health Sciences, University of Gondar, Gondar, Ethiopia; ^4^Institute for Sustainable Futures, University of Technology Sydney, Sydney, NSW, Australia; ^5^Department of Environmental Health, College of Medicine and Health Science, Arba Minch University, Arba Minch, Ethiopia; ^6^Department of Environmental Health, College of Medicine and Health Sciences, Wollo University, Dessie, Ethiopia

**Keywords:** work-related burnout, kindergarten teachers, burnout, children, Ethiopia

## Abstract

**Introduction:**

Work-related burnout is a state of severe physical and mental fatigue and exhaustion resulting from unmanaged prolonged work stress. Kindergarten teachers are at higher risk of work-related burnout compared to other teaching professionals, due to their dual roles as both caregivers and educators for young children. The demands and stresses of these combined responsibilities, coupled with factors such as low salaries, contribute to an increased risk of burnout in this population. Therefore, this study aimed to investigate work-related burnout prevalence and associated factors among kindergarten teachers in Dessie City, Northeast Ethiopia.

**Methods:**

An institution-based, cross-sectional study was conducted in March and April of 2024. A sample of 422 kindergarten teachers was recruited using simple random sampling techniques. A standardized, self-administered Copenhagen Burnout Inventory was utilized to measure work-related burnout. We used EpiData V4.6 and SPSS V26 for data entry and analysis, respectively. A bivariable logistic regression analysis (*p*-value < 0.2) was performed to find factors associated with work-related burnout. Variables found significant in the bivariable analysis were then exported into a multivariable logistic regression model to identify statistically significant variables at a *p* value < 0.05 and a 95% confidence interval.

**Results:**

The response rate was 95% (401/422). 97.5% of the participants were female, and the participants’ mean (standard deviation) age was 28.5 years (SD ± 5.8). In the past 6 months, the total prevalence of WRB was 39.7% [95% CI (34.8, 44.6)]. Work–family conflicts [AOR: 2.81; 95% CI (1.77, 4.45)], working conditions [AOR: 1.63; 95% CI (1.03, 2.56)], perceived stress [AOR: 1.91; 95% CI (1.21, 3.01)], job dissatisfaction [AOR: 1.75, 95% CI (1.10, 2.79)], and kindergarten type [AOR: 1.66; 95% CI (1.02, 2.68)] were factors significantly associated with WRB.

**Conclusion:**

According to this study, a significant number of kindergarten teachers were affected by burnout due to their working nature. To address this issue, interventions focused at reducing work–family conflicts, increasing job satisfaction, and improving the school environment are necessary to support teachers’ well-being, benefiting both young students and the broader educational landscape.

## Introduction

Work-related burnout is a state of severe physical and mental fatigue and exhaustion resulting from unmanaged prolonged work stress (1). It is the result of extensive and prolonged work-related stress, characterized by emotional exhaustion leading to chronic fatigue and a lack of emotional energy; cynicism, which refers to mentally detaching oneself from work and clients; and a reduction in professional performance such as reduced teaching ability, incompetence, and ineffectiveness (2). Work-related burnout (WRB) is a significant public health challenge in the education sector globally. It develops gradually over time, often without the affected individual being fully aware of it (3). Burnout has implications beyond health; it affects a teacher’s passion and commitment and ultimately compromises their ability to meet the needs of their students (4).

Kindergarten (KG) education is an educational process that spans the years from birth to elementary school. It plays a significant role in children’s academic formation, social and emotional development, and health promotion, which requires the physical and mental effort of the teachers in order to provide care and education for children up to the age of primary education (5). It is a specific focus area of Sustainable Development Goals (SDGs), particularly stated under SDG 4, which outlines its objective to “ensure that by 2030, all children have access to quality early childhood care, and pre-primary education”; by doing this, they will be ready for primary school and serve as a foundation for learning and development throughout their lives (6). It is important in terms of providing stimulating environmental opportunities that are appropriate for children’s development and individual characteristics, as well as supporting children’s physical, mental, emotional, and social development (7).

During their preschool period, kids begin to explore, learn, and interact with their surroundings and develop habits and behaviors that fit the social structure of the culture they live in. At this age, KG teachers are essential in giving the intentional guidance that a kid needs at home, at school, and in their social life (8). Mini (9) explained that since today’s children are the citizens of tomorrow’s world, it is necessary to empower them with knowledge and resources to meet their basic needs to enable them to grow to their full potential, as they are the foundation for national development. Therefore, to meet this goal, maintaining the psychological wellbeing of KG teachers is crucial to treating students, making them productive, and being responsible members of the future generation. However, despite Ethiopia is currently giving attention to early childhood development and has created the National Policy Framework, Strategic Operational Plan, and Guidelines for the implementation of high-quality programs for early childhood care and education, the mental health of KG instructors is not well-maintained (10). Presently, the Ministry is also promoting the involvement of private organizations and individuals to fund KG education through missions, non-profits, private individuals, religious institutions, and other organizations that are reopening in urban areas. However, teaching young children in Ethiopia is still regarded as one of the lowest professions due to low pay and unfavorable working conditions that drain their energy and lead to fatigue, which results in teachers losing interest in teaching as a career path and running the risk of burnout. KG teachers work is one of the most demanding and stressful jobs due to their daily responsibilities and frustrations, which often result in physical and mental demands that cause tiredness (5). Their work includes daily hassles like bringing homework, showing up early to school, and staying up late at home, all of which are part of the extremely tough and demanding job that leads to burnout (11).

Apart from this, they have a lot of noticeable and invisible responsibilities that must be fulfilled within a set amount of time that cause them less flexibility and tight work, which is a pertinent cause of burnout (12). The profession by itself needs a responsive caregiver, a competent listener, observer, actor, and topic expert since children who are not physically or mentally mature require the patience and enthusiastic nature of the teachers. However, fulfilling these tasks and managing the emotions accompanying them can be very stressful for early educators, especially if they need the proper training and support (13).

Instructors who experience burnout may be too emotionally exhausted to form close relationships with their students, making it difficult for them to serve as positive role models and communicate with them effectively (14). Children may be bored with learning or not motivated to participate in classroom activities where teachers are exhausted and lack the energy or enthusiasm to encourage their learning, and children may feel the teacher lacks confidence in their teaching (15).

KG teachers’ burnout is thought to have a significant effect on how effectively and successfully they carry out their duties and is reflected on their students, students’ parents, families, and administrators as well (16). This suggests that taking care of teachers’ well-being is not merely an individual concern but has broader implications for the overall educational landscape. Compared to higher-ranking elementary-grade teachers, kindergarten instructors have a yearly turnover rate of 50% or move to another school as a result of working conditions, including burnout at work (17, 18).

From a public health point of view, burnout impacts educators, young children, and the community at large. It caused them lower self-esteem, diminished focus, psychological symptoms like lowered self-control, anger, alienation, anxiety, and depression, and physical symptoms like headaches, stomach issues, elevated blood pressure, sleeplessness, musculoskeletal pain, and increased vulnerability to infections (19, 20). KG teachers WRB is a predictor of performance declines, absenteeism (21, 22), voice disorder (23), hurt teacher-child interaction (24), and reduced students’ autonomous motivation (25). A teacher’s burnout can have a detrimental impact on a number of areas of the classroom environment, including constructive relationships with students or the giving of constructive feedback, pessimism, carelessness, demonization, change resistance, a lack of creativity in instruction, and an unjustified absence (26). The peculiar nature of teaching among KG teachers makes them highly exposed to burnout than other working populations. Prevalence of burnout among KG teachers in China (53.2%) (27), Germany (24.4%) (28), and Greece, KG teachers experience slightly more feelings of emotional exhaustion and depersonalization (29).

Previous research has reported key determinants of WRB such as low job satisfaction (30), KG type (31), working hours per day (32), educational background (33), organizational climate (34), poor or adverse working conditions (35), work stress (36, 37), lack of social support (38, 39), interpersonal conflict (40), and low salaries (27). In addition, previous research has identified various sociodemographic factors associated with burnout among teachers (41–43). For example, female educators are more stressed and burned out than male educators (41, 44). Teachers who are less experienced in their job have a decreased positive work mindset that leads to a serious intention to leave their current employment due to burnout at work (45). Moreover, previous existing literature also shows job stress is positively related to burnout, which means that the higher the stress, the higher the burnout (46). The more teachers gain social support, the less burnout they experience (47) and job satisfaction is protective for burnout (30, 48). Work relationships, role conflict and ambiguity, and a lack of developmental opportunities lead to increased burnout (49). High job demands (50), poor leadership (51), work–family conflicts (52–55), conflict with colleagues, and low decision-making autonomy were factors for burnout (41). However, to date, the issue of WRB has received little attention from the management of the education and teaching sectors.

Work-related burnout is currently the leading occupational health problem affecting service delivery in the education system, and it has become the focus of numerous studies around the world (56). However, it appears that the management of the education and teaching sectors has given little attention to the issue of psychological hazard. There is a paucity of study on Burnout among KG teachers in the study setting and in Ethiopia at large. Moreover, identifying KG teachers’ WRB level helps in increasing their mental and functional performance to satisfy students’ needs during this vital educational stage. Therefore, the purpose of this study was to find WRB prevalence and associated variables among KG teachers in Dessie, northeastern Ethiopia. This study will provide important evidence about the level of burnout and associated variables among KG teachers, enabling the implementation of interventions or policy changes that will enhance teachers’ professional well-being and ultimately improve the quality of education. Moreover, the study’s findings could be pivotal in shaping educational policies and support programs specifically tailored to Ethiopian kindergarten teachers. Given the unique socio-cultural and economic factors in Ethiopia, it is crucial to address the specific challenges faced by educators, particularly in early childhood education.

## Theoretical framework of current study

The theoretical framework of the current study was developed with the help of a literature review. It helped in the extraction of pre-school-level variables and exploring their link with work-related burnout among kindergarten teachers. Different theoretical frameworks that are essentially important for the development and occurrence of burnout were retained to represent the backup theory of burnout (57). Structural theory in occupational health focuses on how an organization’s structure and culture impact employee well-being and burnout. Burnout occurs when rigid organizational structures, unclear roles, low job recognition, inadequate support, and high-pressure cultures and infective coping strategies create excessive stress and emotional exhaustion. This leads to decreased job performance and well-being, necessitating structural changes to support employees effectively (58). Demand-resource theory states that burnout occurs when the demands of work exceed the available resources. This imbalance initially causes fatigue, and if it continues over time, it leads to chronic fatigue and ultimately burnout. For kindergarten teachers, such demands may include work stress, working conditions, managing large class sizes, addressing the emotional needs of young children, and balancing the heavy workload of teaching and administrative responsibilities (59). In contrast, job resources, including job satisfaction, social support, supportive management, access to teaching materials, and a positive work environment, mitigate the impact of these demands, lowering the risk of burnout (60). Social Exchange Theory posits that burnout arises in the social and interpersonal context of the work organization, which is when there is unreciprocated effort at the organizational level or when employees’ expectations regarding the nature of their exchange with the organization are not met (61). Under this theory, perceived value in the exchange can enhance job satisfaction; lack of reciprocity in support may lead to work–family conflicts; work misconduct issues; unmet expectations can cause to intentions to leave; and poor exchanges can contribute to dissatisfaction and burnout. Social cognitive theory: self-efficacy, self-confidence, and self-concept are the three main factors that leads to the evolution and development of burnout, and it arises when an employee has questions about their own or their group’s effectiveness to accomplish work-related goals (58). Under this theory, taking early childhood care training can enhance self-efficacy, impacting how teachers manage stress while their job satisfaction may be affected by their perceptions of their effectiveness and self-concept. By integrating these theoretical perspectives, this study aimed to explore how organizational, work-related and personal factors contribute to burnout among kindergarten teachers. The development of our instrument is grounded in these frameworks, ensuring a comprehensive assessment of both work-related factors [work family conflicts, working condition, job demands and recognition, role clarity, job satisfaction, perceived stress, social support, taking early childhood care training, and individual-level (sociodemographic factors) contributors to burnout].

## Materials and methods

### Study design, period, and setting

An institution-based, cross-sectional study was conducted from March 4 to April 6, 2024, in Dessie, which is a multiethnic city in northeastern Ethiopia. It is located 488 kilometers from Bahirdar, the regional city of Amhara, and 401 kilometers from the capital city of Ethiopia, Addis Ababa. Dessie City has a total of 78 kindergarten schools: 49 private and 29 public. The number of educators in private kindergartens is above 395 and 240 in public kindergartens.

### Population and eligibility criteria

The source population includes all kindergarten teachers in Dessie City and the study population was Teachers who had a minimum of 6 months of experience in the selected KG schools. However, participants who were on sick leave, maternity leave, or annual leave during the time of data collection were excluded in the study.

### Sample size determination and sampling procedure

The sample size was calculated using a single population proportion formula with the following assumptions: Proportion (*p* = 0.5), because no previous study has been conducted among kindergarten teachers in Ethiopia. Margin of error (*d* = 5%) and Z-score ( $Z\alpha {/}_{2}$ = 1.96) corresponding to 95% of the confidence interval. $n={\left(Z\alpha {/}_{2}\right)}^{2}\;\frac{\left[p\;\left(1-p\right)\right]}{d^{2.}}$


$$n={\left(1.96\right)}^{2}\;\left[0.5\;\left(1-0.5\right)\right]/\left(0.05^{2}\right)=384$$

By considering a 10% non-response rate, the total sample size (n) was: *n* = 384* 0.1 + 384 = 38.4 + 384 = 422.

A sample was taken from Dessie City, with a total of 78 kindergarten schools: 49 private and 29 public. For private schools, the number of teachers is 6–24, and for public schools, it is 3–44 per school. We selected 35 private and 25 governmental kindergarten school teachers using simple random sampling. Thirty five selected private kindergarten schools have a total of 315 and 25 selected public schools have a total of 219; the overall number of teachers was 534. A simple random sampling procedure (lottery method) was used to select preschool teachers. To allocate a representative sample in each KG school, a proportional allocation to the size of the school teacher was used.

### Measurement of variables and definition of terms

**Work-related burnout**: was measured using a Copenhagen Burnout Inventory with seven items. The presence or absence of work-related burnout was concluded with the average total score of 50, from which <50 = 0 (no burnout) and ≥ 50 = 1 (burnout) (1, 62).

**Kindergarten teachers**: are professionals who focus on providing care and education to children up to the age of primary education in public, non-public, or for-profit centers or schools that play a significant role in social development and health promotion (5).

**Alcohol drinking**: a kindergarten teacher who drinks any alcoholic drink at least two times per week (63).

**Khat chewing**: a kindergarten teacher who chewed khat three times a week for the last 6 months prior to the study (64).

**Cigarette smoking**: individuals who smoked at least one stick of cigarettes per day for the 6 months prior to the study (65).

**Physical exercise**: a kindergarten teacher who performed physical exercise at least two times per week for 30 min (66).

**Job demand** and role conflict with four items; job recognition and role clarity with three items on a 5-point Likert scale and dichotomized by their mean (67).

**Job satisfaction:** 10 items from a general job satisfaction scale score of 10–31 were satisfied, and 32 or above were dissatisfied (68).

**Social support** was measured by a 3-item Oslo Social Support Scale in which poor (3–8), moderate (9–11), and strong social support (12–14) (69).

**Work–family conflicts** were measured with 10 items on a seven-point Likert scale and dichotomized by mean (70).

**Working conditions** were measured by five items related to the conduciveness of class room conditions and school facilities (staff rooms, recreation centers, toilets, internet access, etc.), collegial relationships among teachers, and the number of kids in a class room, and dichotomized by their mean value (71).

**Perceived work stress** was measured by 14 items with five Likert scales and dichotomized as greater than or equal to 28 high and below 28 as low (72).

**Turnover intention** was measured by six items on a five-point Likert scale and dichotomized as below 18, which indicates an intention to stay, while a total score above 18 indicates an intention to leave (73).

### Data collection tools and procedures

An anonymous, standard, self-administered questionnaire was used to collect the data. The development process of these instruments began with the selection of constructs, with a thorough literature review as measuring constructs relevant to this study. After that, each instrument’s items were carefully selected based on their relevance to the specific objective of this study. The selection criteria are aligned with the theoretical frameworks. Once suitable instruments are identified, we have adapted to fit the cultural, social, and linguistic context of the study to our study population. Then the number of items from each instrument was chosen to measure specific constructs and communicating with health professionals, school teachers, and kindergarten teachers during the pretest to ensure the relevance of the questioner to measure the intended outcomes. Moreover, to ensure the tools’ reliability, we test internal consistency of each construct.

### The Copenhagen burnout inventory

The CBI was employed to evaluate WRB, which has been used and verified in previous research (74, 75). It is a universal tool that has been translated into multiple languages and is presently being utilized in numerous countries to assess risk factors related to the workplace (1). The CBI classifies burnout into three subscales, such as burnout related to personal, work, and client (76, 77). However, in the current study, only work-related burnout was investigated. WRB was made up of seven items. The validity and dependability of CBI in Africa were examined among Nigerian resident physicians, and the results revealed the highest internal consistency (78). This instrument is locally tailored to Ethiopia and has demonstrated excellent dependability in earlier research (79, 80). The instrument’s response format and scoring system are as follows: each of the seven elicited items has a five-point Likert scale, with very low/never = 1, low/rarely = 2, somewhat/sometimes = 3, high/often = 4, and very high/always = 5. Following the data collection, for negative items, each Likert scale label was reverse-coded using one of the following formats: 1 = 5, 2 = 4, and 3 = 3. Then each Likert scale label was recoded using the following formats: 1 = 0, 2 = 25, 3 = 50, 4 = 75, and 5 = 100, and then all the items were summed. Then, each choice was given one of the following weighted percentages: never (0%), seldom (25%), sometimes (50%), often (75%), and always (hundred percent) (77, 81). In this study the internal dependability (Chronbach’s alpha) was 0.82.

### The Copenhagen psychosocial questionnaire

It is a standardized universal instrument with a five-point Likert scale intended to evaluate workplace health promotion and psychosocial circumstances (67). It is free and available to the public and can be applied to any kind of workplace. In Ethiopia, these tools are customized and extensively used in different working groups with good dependability (79, 80). We use the job demands scale from the Copenhagen Psychosocial Questionnaire to quantitatively assess specific aspects of the demands faced by workers (79, 80). These four items help identify challenges such as whether workloads are unevenly distributed or workers feel they are falling behind and how well they can manage their time. Through rigorous quantification of these dimensions, the tool enables a clear assessment of how these job demands contribute to overall stress and burnout levels. In this study, the scale has Cronbach’s alpha was 0.84.

### Perceived stress scale

A standardized PSS questionnaire was applied to measure the perceived work stress (72). It is a brief and simple tool with significant validity and reliability to measure the degree to which the conditions in one’s life are perceived as stressful for the genesis of illness and behavioral disorders. The 14 items on the scale range from never = 0, almost never = 1, sometimes = 2, fairly often = 3, and very often = 4 were selected based on their relevance to the specific objective of this study. To achieve the tool’s scores, the scores of seven positive items (four, five, six, seven, nine, ten, and thirteen) are reversed, such that 0 = 4, 1 = 3, 2 = 2, and so on, and then the 14 items are added up (72). This instrument is customized and tailored in Ethiopian cultures and used in various studies, and it was confirmed that the tool is reliable (82, 83). Moreover, the reliability (Chronbach’s alpha) of PSS in this study was 0.77.

### Job satisfaction

Job satisfaction was evaluated using a 10-item generic work satisfaction scale developed by MacDonald and MacIntyre (68). This scale, validated in Canada across various occupations and organizations, has proven to provide reliable and valid scores (68). In Ethiopia, the scale has been tailored into local languages and applied to diverse working populations, including school teachers, where it has also demonstrated excellent reliability (80, 84). The internal reliability of 10 items of job satisfaction scale has a Chronbach’s alpha of 0.90.

### Work–family conflicts scale

Work–family conflicts (WFCs) are key factors in occupational health, linked to reduced organizational commitment, poor job performance, and various negative outcomes like burnout, depression, and sleep issues. In this study, WFCs were evaluated using a 10-item self-report questionnaire that includes two scales: work–family conflict and family–work conflict. These scales are widely recognized for their reliability and validity across different professions and countries (70). In this study, a pretest was done to ensure clarity and cultural relevance of items for sensitivity to Ethiopian norms regarding work and family roles, and feedback from local educators helped modify language for better relevance. In addition, the internal reliability of Work–family conflicts 10 items has Chronbach’s alpha of 0.86.

### Working conditions scale

Working conditions scale (WCS) was adapted from previous literature to assess the conduciveness of class room conditions and school facilities (staff rooms, recreation centers, toilets, internet access, etc.), collegial relationships among teachers, and the number of kids in a class room, and dichotomized by their mean value (71). In the present study, the scales Cronbach’s alpha was *α* = 0.89.

### Turnover intention scale

Turnover intention was (TIS) assessed using six items on a five-point Likert scale, indicating a desire to leave the job (73). In the current study, the scales Cronbach’s alpha reliability (α = 0.907) demonstrating high internal consistency.

### Social support questionnaire

The three-item Oslo Social Support Questionnaire evaluates psychosocial resources within social networks. It’s a brief, cost-effective tool used in various large-scale surveys, including the European Opinion Research Group (2003), Ireland’s National Lifestyle Survey (2007), the European Kid Screen Study, and the Outcome of Depression European Network study (69). This instrument has been tailored and utilized in several studies conducted in Ethiopia, which confirm that the tool is reliable (85–87). In this study social support three items scale has a Chronbach’s alpha values of 0.86.

The final versions of the questionnaire comprise four sections. The first section comprises socio-demographic characteristics, which assess information on age, sex, educational level, work experience, having children, monthly salary, working hour per day, and types of kindergarten. The second category encompasses questions to assess information on work-related burnout. The third part of the questionnaires includes behavioral factors like cigarette smoking, khat chewing, alcohol drinking, medication use for stress relevance, work misconduct issues, and physical exercise, and the fourth question assessed different factors in the workplace, such as work–family conflicts, job satisfaction, perceived stress, social support, working conditions, and the Copenhagen psychosocial questionnaire (62).

### Data quality control

To ensure consistency, the survey instrument was first developed in English, translated into Amharic, the native tongue, and then back to English by language specialists. The questionnaires were pre-tested and structured. The pre-test was done on 5% of the total sample that was excluded in the final analysis. The reliability test from Chronbach’s alpha was measured and calculated. The data were collected by two BSc-level environmental health professionals. One supervisor was recruited for continuous supervision of the data collectors. Data collectors and a supervisor received 2 days of training covering the goals of the study, data collection methods, and ethical guidelines. Every day, the lead investigator and supervisor reviewed the gathered data to ensure it was accurate, clear, and comprehensive, and any necessary changes were made immediately. Data cleansing and cross-checking were completed prior to analysis.

### Data management and analysis

The data were imported into Epi-data version 4.6, exported, and cleaned before being analyzed using SPSS version 26 after accuracy tests. Cross-tabulations were conducted to evaluate the link between the outcome variable (WRB) and its associated factors, and frequency tables, and percentages were utilized to provide descriptive statistics. To handle confounding, we apply random sampling and multivariable regression analysis, and validated instruments were used to collect comprehensive data within a conceptual framework. The variables’ multicollinearity, outliers, and normality were assessed before the bivariable and multivariable binary logistic regression analyzes were performed. The variance inflation factor was used to verify the multicollinearity assumption (VIF < 2). In the bivariable logistic regression analysis, variables demonstrating statistically significant correlations with the dependent variable at *p*-values < 0.2 were retained for further analysis. Using this *p*-value threshold is appropriate for preliminary screening to identify potential associations between the independent variables and the outcome of interest. This approach is valuable to avoid missing potentially important variables that could have significant implications for public health or policy. This higher *p*-value threshold helps ensure that potentially significant associations are not overlooked during this initial analysis phase. The model’s fitness was checked by the Hosmer and Lemeshow goodness-of-fit (*p* = 0.802), and the results showed that it was well fitted (*p* value > 0.05). Finally, factors with a *p*-value < 0.05 were found using a multivariable binary logistic regression model. A 95% confidence interval-based adjusted odds ratio (AOR) was used to show the degree of relationship.

## Results

### Socio-demographic characteristics of participants

From a total of 422 selected KG teachers, 401 of them participated, with a response rate of 95%. The mean (±SD) age of the study participants was 28.5 (±5.8). The majority of the participants were female [391 (97.5%)], 240 (59.9%) were married, and 213 (53.1%) had at least one child. Regarding their educational level, 184 (45.9%) of them were diploma holders, and 87 (21.7%) of them had certificates. 174 (43.4%) of the participants had less than 5 years of teaching experience; 109 (27.2%) of them had less than a 3,000 Ethiopian birr (ETB) monthly salary; and the majority of 261 (65.1%) of them were taking early childhood care trainings (Table 1).

**Table 1:** Socio-demographic characteristics of kindergarten teachers in Dessie City, Northeast Ethiopia, 2024 (*n* = 401).

Variables	Frequency (*n*)	Percent (%)
Sex		
Male	10	2.5
Female	391	97.5
Age		
21–29	171	42.6
30–38	102	25.4
39–47	80	20.0
≥48	48	12.0
Religion		
Orthodox	239	59.6
Muslim	145	36.2
Protestant	17	4.2
Marital status		
Single	131	32.7
Married	240	59.9
Divorced	25	6.2
Widowed	5	1.2
Having children		
No children	188	46.9
At least one children	213	53.1
Number of children		
1–2	152	37.9
3–6	61	15.2
Educational level		
Certificate	87	21.7
Grade 11 and 12 complete	67	16.7
Diploma	184	45.9
Bachelor’s Degree	63	15.7
Work-experience in years		
<5	174	43.4
5–10	113	28.2
11–15	61	15.2
≥16	53	13.2
Weekly class schedule		
1–4	42	10.5
5–6	359	89.5
Daily working hour		
≤8	369	92.0
>8	32	8.0
Monthly salary in Ethiopian birr		
1,609–3,000	109	27.2
3,100–4,000	98	24.4
4,001–5,100	95	23.7
5,101–10,000	99	24.7
Kindergarten type		
Public	185	46.1
Private	216	53.9
Taking early childhood care training		
Yes	261	65.1
No	140	34.9

### Behavioral characteristics of participants

Of the participants, only 25 (6.2%) were alcohol drinkers, and none of them were smokers. The majority of participants were doing physical exercise for at least 2 days per week for 30 min. Among the study participants, 99 (24.7%) have work misconduct issues that may probably cause burnout in their professions. Only 57 (14.2%) of them used medication for stress related to their work (Table 2).

**Table 2:** Behavioral characteristics of kindergarten teachers working in Dessie City, Northeast Ethiopia, 2024 (*n* = 401).

Variables	Frequency (*n*)	Percent (%)
Alcohol drinking		
Yes	25	6.2
No	376	93.8
Khat chewing		
Yes	4	1.0
No	397	99.0
Medication use		
Yes	57	14.2
No	344	85.8
Doing physical exercise		
Yes	263	65.6
No	138	34.4
Having work misconduct issue		
Yes	99	24.7
No	302	75.3

### Work-related characteristics of kindergarten teachers in Dessie City

In terms of work-related characteristics, 190 (47.4%) had high job demands, and only 38 (9.5%) had low social support. Among them, 199 (49.6%) of them have work–family conflicts, and 220 (54.9%) of them work in poor working conditions. In terms of their role, 139 (34.7%) of them have poor role clarity, 190 (47.4%) were stressed, and 217 (54.1%) have a high turnover intention of leaving their job (Table 3).

**Table 3:** Work-related characteristics of kindergarten teachers in Dessie City, Northeast Ethiopia, 2024 (*n* = 401).

Variable	Frequency (*n*)	Percent (%)
Job demand		
High	190	47.4
Low	211	52.6
Job recognition		
High	272	67.8
Low	129	32.2
Role clarity		
Good	262	65.3
Poor	139	34.7
Social support		
Low	38	9.5
Moderate	175	43.6
High	188	46.9
Work–family conflicts		
High	199	49.6
Low	202	50.4
Working conditions		
Good	181	45.1
Poor	220	54.9
Job Satisfaction		
Satisfied	164	40.9
Dissatisfied	237	59.1
Perceived Stress		
Stressed	190	47.4
Not Stressed	211	52.6
Turnover intention		
High	217	54.1
Low	184	45.9

### Prevalence of work-related burnout

The total prevalence of WRB in the last 6 months was 39.7% (*n* = 159) [95% CI (34.8, 44.6)]. Table 4 displays the findings of all seven WRB items, together with the corresponding frequency and mean scores.

**Table 4:** Response categories and scoring system of work-related burnout among kindergarten teachers in Dessie City, Northeast Ethiopia, 2024 (*n* = 401).

Work-related burnout items (Chrombach’s alpha = 0.82)	Response category and scoring system					
	Never scoring	Seldom scoring	Some times scoring	Often scoring	Always scoring	Score mean (SD)
	0%	25%	50%	75%	100%	
1. Is your work emotionally exhausting?	11.2	29.9	29.9	11.5	17.5	48.50 (31.24)
2. Do you feel burnout because of your work?	16.5	35.4	26.2	12.0	10.0	40.90 (29.60)
3. Does your work frustrate you?	31.4	30.9	24.2	8.7	4.7	31.11 (28.23)
4. Do you feel worn out at the end of the working day?	13.5	29.9	28.2	13.2	15.2	46.70 (31.27)
5. Are you exhausted in the morning at the thought of another day at work?	34.2	27.4	24.2	11.2	3.0	30.36 (28.05)
6. Do you feel that every working hour is tiring for you?	20.4	31.7	27.2	14.0	6.7	38.72 (28.97)
7. Do you have enough energy for family and friends during leisure time? (Reversed scoring)	1.7	7.2	28.4	31.4	31.2	70.76 (25.20)
Total average score						43.86
						28.94

### Factors associated with work-related burnout

In the bi-variable logistic regression analysis, educational level, kindergarten type, participant age, job demand, working condition, perceived stress, job satisfaction, work–family conflicts, work misconduct issue, work experience, and taking early childhood care training were factors associated with WRB (*p* < 0.2). Finally, after adjusting for confounding variables in the multivariable binary logistic regression analysis, only kindergarten type, job satisfaction, working conditions, perceived stress, and work–family conflicts were discovered to be statistically significant variables associated with WRB (*p*-value < 0.05).

As a result, KG teachers who reported high work–family conflicts had a 2.8 times higher chance of developing WRB than those with low work–family conflicts [AOR: 2.81; 95% CI (1.77, 4.45)]. KG teachers who were not satisfied with their jobs showed a 1.8-fold increased risk of WRB compared to those satisfied with their job [AOR: 1.75, 95% CI (1.10, 2.79)]. Stressed KG teachers were 1.9 times more likely to suffer WRB than those who are not stressed [AOR: 1.91; 95% CI (1.21, 3.01)]. Compared to their peers’, those who work under poor working conditions had a 1.6 times higher risk of experiencing work-related burnout [AOR: 1.63; 95% CI (1.03, 2.56)]. Teachers working in public KG schools had a 1.7-fold increased risk of getting WRB than their counterparts’ [AOR: 1.66; 95% CI (1.02, 2.68)] (Table 5).

**Table 5:** Bi-variable and multivariable binary logistic regression analysis of associated factors with work-related burnout among kindergarten teachers in Dessie City, Northeast Ethiopia, 2024 (*n* = 401).

Variables (*N* = 422)	Work-related burnout		COR with 95% CI	AOR with 95% CI
	Yes	No		
Age				
21–29	78	93	1.68 (0.86, 3.28)	1.46 (0.68, 3.16)
30–38	46	56	1.64 (0.80, 3.36)	1.96 (0.83, 4.60)
39–47	19	61	0.62 (0.28, 1.37)	0.57 (0.24, 1.39)
≥48	16	32	1	1
Work experience in year				
<5	86	88	2.26 (1.17, 4.36)	1.84 (0.88, 3.88)
5–10	43	70	1.42 (0.71, 2.86)	0.93 (0.42, 2.06)
11–15	14	47	0.69 (0.30, 1.59)	0.53 (0.21, 1.36)
≥16	16	37	1	1
Kindergarten type				
Public	88	97	1.85 (1.24, 2.78)	1.66 (1.02, 2.68)^*^
Private	71	145	1	1
Work misconduct issue				
Yes	46	53	1.45 (0.92, 2.30)	1.47 (0.85, 2.54)
No	113	189	1	1
Early childhood care training				
Yes	97	164	1	1
No	62	78	1.34 (0.89, 2.04)	1.29 (0.80, 2.09)
Job satisfaction				
Dissatisfied	107	130	1.77 (1.17, 2.69)	1.75 (1.10, 2.79)^*^
Satisfied	52	112	1	1
Perceived stress				
Stressed	90	100	1.85 (1.24, 2.78)	1.91 (1.21, 3.01)^*^
Not stressed	69	142	1	1
Job demand				
High	83	107	1.38 (0.92, 2.06)	1.44 (0.92, 2.27)
Low	76	135	1	1
Work–family conflicts				
High	100	99	2.45 (1.62, 3.69)	2.81 (1.77, 4.45)^**^
Low	59	143	1	1
Working conditions				
Poor	98	122	1.58 (1.05, 2.37)	1.63 (1.03, 2.56)*
Good	61	120	1	1

Crude odds ratio (COR); confidence interval (CI); adjusted odds ratio (AOR); reference category (1); **statistically significant at *p* < 0.001; *statistically significant at *p* < 0.05; Hosmer and Lemeshow test = 0.802; it showed that the model fitted well.

## Discussion

This study aimed to investigating the prevalence of WRB and its associated factors among kindergarten teachers. As a result, the total prevalence of WRB in the last 6 months was 39.7% [95% CI (34.8, 44.6)]. This indicates that burnout level among kindergarten teachers in Dessie City, Ethiopia, was high. In addition, work–family conflicts, working conditions, perceived stress, job satisfaction, and kindergarten type were factors significantly related to WRB. This study’s findings are grounded in structural theory that illustrates how an organization’s structure and culture impact employee well-being, and an unmanaged stress level causes burnout (58). In addition, the findings of this research are supported by demand-resource theory that posits burnout occurs when the demands of work, such as high perceived stress and poor working conditions, lead to chronic fatigue and ultimately burnout (60). Moreover, the results of this study are grounded in Social Exchange Theory, which explains burnout arises in the social and interpersonal context of the work organization when there is a lack of reciprocity at the organizational level or when employees’ expectations regarding the nature of their exchange with the organization are not met, which creates work–family conflicts and makes them dissatisfied with their job (61).

The prevalence of WRB in this finding was almost similar to studies conducted among school teachers in Brazil (36.7%) (88), Nigeria (36%) (89), and Ethiopia (37.4%) (79). This resemblance could be explained by the long working hours and intense nature of the teaching profession, which contribute to chronic stress and increases the risk of burnout (46). However, the current study finding is higher as compared with kindergarten teachers in Germany (24.4%) (28), and school teachers in Iraq (24.5%) (90), Namibia (28.8%) (91), and Tunisia (27.4%) (35). The possible reason for this might be kindergarten teachers need to put in sufficient physical and psychological effort to accomplish their work, but KG teachers in Dessie City gain significantly lower recognition, a low salary, and low social-economic status, plus their work nature leads to high burnout. Besides, their salary is insufficient to administer their lives; they have a dual role as caregiver and educator, which may exhaust their energy and cause burnout (92). In addition, their work is demanding due to the nature of the children, who are immature, and they are always in touch with children, and their role is not limited to teaching and conveying information to children but instead has a role with various aspects and characteristics as a role in developing the educational process. Furthermore, kindergarten teachers having a long working hour, high intensity, and wide range of emotional interactions (93) with their students causes them stress, fatigue, emotional exhaustion, and more burnout, which decreases teaching performance and leads to less interaction with students in their classrooms (14). Other possible reasons may be variations in study settings, tool differences, and methods of data collection. In contrast, this study indicated a decreased prevalence of WRB compared to studies in China (27). This may be due to cultural differences, variations in measurement tools, and the specific contexts in which the teachers work.

This study found that work–family conflicts were associated with WRB. This finding is supported by studies in India (94), Indonesia (52), Portugal (53), Hungary (54), Malaysia (55), and China (34). This might be because the majority, or all, of the KG teachers were female teachers. They performed dual roles, managing both their job responsibilities and family care, and experienced strain from work demands that interfered with their home responsibilities. This ultimately led to the spillover of high work demands into their home life, causing depletion of energy and contributing to job burnout (55). WFCs affects work engagement by inducing stress, tension, sleep disturbances, and other adverse psychological and physical symptoms that trigger the energy exhaustion of KG teachers and lead to burnout (95, 96). If kindergarten teachers are unable to fulfill the expectations of their families and employers, this condition may lead to disputes and disagreements that can subsequently result in mental pressure and experiences of burnout (97). Another possible reason is the prolonged working hours of KG teachers, which are 9 h and sometimes 10 h in school per day. This extensive time spent at work can lead to neglecting household responsibilities and family care, exacerbating WFCs. When challenges at home and in their social lives hinder teachers’ ability to fulfill work obligations, it creates family–work conflict, resulting in time pressures, reduced flexibility, fatigue, and energy depletion. Consequently, teachers may devote less time to their families, which can intensify WFCs and ultimately lead to burnout (98). Additionally, the cost of living rising in Ethiopia places further burdens on teachers, compelling them to manage their professional, social, and familial responsibilities simultaneously, thus contributing to WFCs and increasing the risk of stress and burnout.

In this study, job dissatisfaction was associated with WRB. This finding is corroborated in studies conducted in Malaysia (99), Norway (100), and Italy (30). This is due to the fact that job dissatisfaction is associated with high levels of burnout (101). In addition, job satisfaction, which is the overall feeling about one’s job or career, is used as a buffer against burnout, and their job satisfaction has implications for student learning in that a satisfied teacher may provide better quality or more consistent instruction to his or her students. Furthermore, teachers with low salaries and poor working conditions may lose their emotional resources and job satisfaction, leading to energy depletion and increased susceptibility to burnout. Conversely, when teachers experience a decline in job satisfaction, it can heighten feelings of cynicism and reduce their sense of personal accomplishment, which may further contribute to burnout (102).

In addition, the findings of this study highlight that working conditions are statistically associated with WRB. This finding is concurrent with findings in Tanzania (103), Iraq (90), Sweden (51), and Brazil (104). Possible reasons for this correlation might be the presence of factors such as inadequate school facilities, work overload, a high number of students per class, and poor staff relations. KG teachers working under these challenging conditions may feel pressured and stressed, consequently increasing their likelihood of developing WRB. Besides, a highly demanding work atmosphere can lead to strain reactions in teachers and impair their work performance, making it one of the key factors related to burnout. Moreover, KG teachers face numerous challenges in the development of society and education, which contribute to increased workloads. These challenges include curriculum adaptation, the use of diverse educational tools, and effective classroom management. When employees are unable to cope with these demanding working conditions, they may feel restless, fatigued, exhausted, and ultimately experience burnout. Furthermore, the short breaks between classes contribute to the exhaustion felt by KG teachers, as they are under significant pressure from parents and their expectations. This pressure can lead teachers to experience detachment, emotional exhaustion, and cynicism (34). Another likely reason is that KG teachers’ work under conditions that require them to take charge of children’s safety and care, which can lead to high job stress. This stress often contributes to negative emotional issues such as despair, anxiety, and burnout (105).

Moreover, in this study, perceived workplace stress is associated with burnout. This finding is in agreement with the findings in Japan (106), China (27, 107), and the South Central United States (108). One possible explanation is that educators who experience high levels of stress are having a high chance of developing WRB (109). The challenging and unique nature of their work, including managing crying children, dressing and undressing them, overseeing lunchtime and clean-up, monitoring nap times, and continuous exposure to germs and illness, contributes to this stress. Additionally, the lack of enough breaks during their workday and unclear delineation of work duties can lead to frustration and heightened stress, ultimately resulting in burnout (110). Another likely explanation is that they may experience heightened perceived stress due to a demanding workload, the number of kids per classroom, lack of resources, poor pay, low job status, numerous classroom responsibilities, curriculum demands, testing, performance evaluations, disruptive students, and the burden of providing direct care for preschool children and meeting their needs (59). These factors are significant contributors to stress and ultimately lead to burnout (111, 112). Moreover, the peculiar nature of their work, such as providing verbal and visual support, offering repeated explanations, assisting with the development of social skills, orienting children in space and time, resolving conflicts, facilitating communication and social relationships, supervising rest periods, and preparing teaching aids and materials can contribute to stress that depletes their energy and ultimately leads to burnout (113). Lastly, kindergarten teachers must develop creative ways to make their classes engaging and visually appealing. Some of these strategies include role plays, singing, dancing, interactive games, creating an interactive stage, drawing, and offering prize incentives. However, while these activities can enhance the learning environment, they can also become sources of stress and exhaustion for teachers. If they are unable to manage these demands, it may ultimately lead to burnout (114).

Lastly, in this study, public kindergarten teachers had more burnout as compared to their private counterparts. This finding is supported by a study in China (27). The possible explanations might be that private kindergartens are often newer, have smaller class sizes, and their head instructors have a high chance of receiving training in early childhood care compared to public kindergartens (115). Additionally, kids enrolled in private kindergartens typically come from wealthier families and tend to score higher on school readiness assessments. This readiness is essential for facilitating the teaching and learning process, which may reduce the burden on teachers (115). On the contrary, public kindergarten teachers were less likely to get the chance of receiving early childhood care training, which may result in lower scores on emotional support and behavior management in their classrooms (108). This is evidenced by our observations during data collection, which highlighted that in a public kindergarten, there were a large number of children in one class, with insufficient sleeping space during nap time. This overcrowding can lead to behavioral disturbances and create challenges for the teachers. Moreover, lack of facilities such as separate mail areas, hand washing stations, and adequate playgrounds with necessary materials increased the burden on teachers to address these gaps, ultimately leading to heightened stress and difficulties in supporting the children effectively. On top of that, there are differences in the required qualifications and work environments between public and private kindergartens. Educators in private schools have autonomy, administrative support, and minimal bureaucratic layers; they tend to be more professionally certified and may be less likely to experience burnout (116). Conversely, while teachers in public school are regarded as public servants and are guaranteed jobs until retirement, along with a pension after retiring, they face significant challenges. These include high workloads, insufficient salaries, low recognition and promotion opportunities, limited resources that hinder the teaching-learning process, and unsatisfactory working conditions. These factors can intensify the level of burnout among public kindergarten teachers (117).

### Strengths and limitations of the study

This study used a standardized and reliable tool with a sufficient sample size from several kindergarten schools, including both public and private, and can be used as baseline information for programmers and other researchers. In addition, this study may contribute to the growing corpus of research on WRB and its predictors in this population. However, the study was based on a cross-sectional study design, which hinders the temporal relationship between WRB and factors affecting its development. Moreover, since the source data were self-reported from prior experiences of kindergarten teachers, recall and social desirability bias may underestimate or overestimate the level of burnout. To reduce this bias, we restrict the data to recent experiences only. The study focuses on kindergarten teachers in a specific city, which might overlook factors that could be relevant in other educational levels or geographical areas within the country. To demonstrate the association between different workplace aspects and WRB, future research must take into account a range of workplaces. With the exception of these limitations, we think this study provides trustworthy and robust data to address kindergarten teachers’ work-related burnout in Dessie, Ethiopia.

### Implications of the study

The study’s findings are crucial in shaping educational policies and support programs tailored to Ethiopian kindergarten teachers, considering the unique socio-cultural and economic factors in the country. The study advocates for policies that prioritize teacher well-being as a key component of educational quality. This could include regular mental health assessments and support integrated into the national teacher support framework. The findings suggest implementing professional development programs focused on stress management, work-life balance, working conditions, job satisfaction, and coping strategies designed specifically for kindergarten teachers. These could be integrated into existing teacher training or offered as ongoing professional development. In addition, the research recommends creating localized teacher support networks such as strategies for improving teacher-student ratios, financial incentives, stress management workshops allowing teachers to share experiences, seek peer support, and access resources to manage burnout. These networks could be supported by local educational authorities. Moreover, this study suggests long-term policy recommendations, such that the study calls for the establishment of long-term monitoring and evaluation mechanisms to continuously assess teacher burnout. This could lead to a national framework for teacher well-being aligned with Ethiopia’s broader educational goals.

## Conclusion

According to this study, a significant number of kindergarten teachers were affected by burnout due to their working nature. KG teachers’ WRB was found to be predicted by factors such as work–family conflicts, job satisfaction, perceived stress, kindergarten type, and working conditions. Interventions focused at reducing work–family conflicts, increasing job satisfaction, and improving the school environment are needed to address this critical issue and support teachers’ well-being for the benefit of young students and the wider educational landscape.

## Data Availability

The original data is provided in the article, and further inquiries are directed to the corresponding author upon request.
